# Price, Promotion, and Availability of Nutrition Information: A Descriptive Study of a Popular Fast Food Chain in New York City

**DOI:** 10.5539/gjhs.v5n6p73

**Published:** 2013-08-25

**Authors:** Corey Hannah Basch, Danna Ethan, Sonali Rajan

**Affiliations:** 1 Department of Public Health, William Paterson University, United States; 2 Department of Health Sciences, Lehman College, The City University of New York, United States; 3 Department of Health and Behavior Studies, Teachers College, Columbia University, United States

**Keywords:** fast food, nutrition information, promotional strategies, pricing

## Abstract

Legislation in NYC requires chain restaurants to post calorie information on menu boards in an effort to help consumers make more informed decisions about food and beverage items they are purchasing. While this is a step in the right direction in light of the current obesity epidemic, there are other issues that warrant attention in a fast food setting, namely the pricing of healthy food options, promotional strategies, and access to comprehensive nutrition information. This study focused on a popular fast-food chain in NYC. The study’s aims were threefold: (1) to determine the cost differential between the healthiest meal item on the chain’s general menu and meal items available specifically on a reduced cost menu for one dollar (US$1.00); (2) to identify and describe the promotions advertised in the windows of these restaurants, as well as the nutrition content of promoted items; and (3) to ascertain availability of comprehensive nutrition information to consumers within the restaurants. We found the healthiest meal item to be significantly higher in price than less nutritious meal items available for $1.00 (t = 146.9, p < .001), with the mean cost differential equal to $4.33 (95% CI $4.27, $4.39). Window promotions generally advertised less healthful menu items, which may aid in priming customers to purchase these versus more healthful options. Comprehensive nutrition information beyond calorie counts was not readily accessible prior to purchasing. In addition to improving access to comprehensive nutrition information, advertising more of and lowering the prices of nutritious options may encourage consumers to purchase healthier foods in a fast food setting. Additional research in this area is needed in other geographic locations and restaurant chains.

## 1. Introduction

The Patient Protection and Affordable Care Act of 2010 requires chain restaurants to post calorie information on menus (Govtrack.us, 2010; Nestle, 2010). New York City (NYC) began this practice in 2008 per Section 81.50 of the NYC Health Code (NYC.gov, 2008). In light of the obesity epidemic (Ogden, Carroll, Kit, & Flegal, 2012; Flegal, Carroll, Ogden, & Johnson, 2002; Ogden, Flegal, Carroll, & Johnson, 2002), these policies were implemented as an effort to counter the current obesogenic environment and provide more information to consumers as they make decisions about meals they are purchasing (National Conference of State Legislatures, 2013). Given the popularity of fast food and the prevalence of fast food chains in neighborhoods where rates of overweight and obesity are particularly high, this policy has numerous public health implications (Anderson, Rafferty, Lyon-Callo, Fussman, & Imes, 2011; Fleischhacker, Evenson, Rodriguez, & Ammerman, 2011; Bodor, Rice, Farley, Swalm, & Rose, 2010). Further, given that the nutritional quality of fast foods has been shown to be consistently poor (Kirkpatrick et al., 2013), efforts to minimize the obesogenic nature of fast food environments in particular are crucial.

Studies conducted in NYC on the effectiveness of menu labeling in helping consumers reduce calorie intake by choosing lower-calorie options have had mixed results. Consumer awareness and use of calorie information were assessed in two studies where customers who reported seeing this information were found to be more likely to use this to inform their purchase (Dumanovsky, Huang, Bassett, & Silver, 2010; Bassett et al., 2008). Conversely, a commonly cited study found that calorie labeling had no impact on calories consumed in select fast food restaurants among either adults or children (Elbel, Rogan, Brescoll, & Dixon, 2010; Elbel, Gyamfi & Kersh, 2011). Further research has identified specific barriers to utilizing menu labeling, including cost of the items, being pressed for time, and not understanding the posted calorie information (Schindler, Kiszko, Abrams, Islam, & Elbel, 2013).

Earlier research has assessed availability of nutrition information within fast food establishments (Wootan & Osborn, 2006; Wootan, Osborn, & Malloy, 2006) and also identified how frequently consumers examined available nutrition information (Roberto, Agnew, & Brownell, 2009). However, since the posting of caloric information began in NYC in 2008, less attention has been devoted to specifically examining access to more comprehensive nutrition information in these establishments. At the same time, efforts have been made to include healthier food options at these establishments. Some have taken steps to offer more healthful food and beverage choices to its customers. However, given that price has been previously cited as being a barrier to making healthier food choices (Schindler et al., 2013), the need to identify the cost of these healthier items, particularly in comparison to other heavily promoted menu items, is critical.

A key component of commonly used marketing practices for businesses is promotion, which entails strategizing ways in which demand for a product can be increased or differentiated (Goi, 2009). A recent report on promotional strategies used in fast food chains indicated that 80% of restaurants had promotions posted on the exterior of the building (Bridging the Gap, 2012). Further, the products being promoted were found to be primarily high in calories and low in nutritional value (Bridging the Gap, 2012). Additional study findings also suggest that low-income populations are most commonly exposed to outdoor advertisements of this nature (Grier & Kumanyika, 2008; Yancey et al., 2009). Indeed these promotions have been noted for advocating increased consumption of nutrient-poor and calorie-dense foods (Seiders & Petty, 2004; Larson & Story, 2009), and as such appear to contribute to the obesogenic nature of local neighborhood environments.

## 2. Material & Method

### 2.1 Material Studied

The aims of the present study were threefold: 1) to determine the cost differential between the healthiest meal item on the chain’s general menu (a Caesar salad with grilled chicken) and the meal items specifically available on the chain’s reduced cost menu for one dollar (US$1.00); 2) to identify and describe the promotions advertised in the windows of these restaurants, as well as the nutrition content of the promoted items; and 3) to ascertain the availability of comprehensive nutrition information to consumers within the restaurants. Implications for the obesity epidemic in NYC are discussed and specific recommendations to promote customers’ informed decision-making and more healthful food choices in a fast food setting, particularly in boroughs where the rates of obesity are highest, are presented.

#### 2.1.1 Area Descriptions

We sought to identify restaurants of a popular fast food chain in the two highest-income NYC boroughs (Manhattan and Staten Island) and those in the two lowest-income boroughs (Bronx and Brooklyn) (Kopans, Miceli, Petillo, & Venkatesh, 2007). The Bronx and Brooklyn are also the two boroughs with the highest rates of overweight and obesity in NYC (Kopans et al., 2007) and are therefore of particular interest as we seek to understand environmental factors that may contribute to the progression of the obesity epidemic. We compiled a list of food chain locations from their company’s online search engine using the following search terms: Bronx, Brooklyn, Manhattan, Staten Island, and New York, NY.

### 2.2 Methods

#### 2.2.1 Study Design

This was a descriptive study. Data were intentionally collected at one point in time to avoid possible fluctuations in price and similarly to ensure that the same food and beverage products were available at all restaurants in the sample.

#### 2.2.2 Sampling Frame

Restaurants were excluded from our sampling frame for not selling the Caesar salad with grilled chicken (n = 4), for being located in an atypical location such as an airport or bus terminal or a shopping mall (n = 5), and/or for not having a working telephone number after a search from directory assistance could not provide a new number (n = 27). Our final sampling frame consisted of 140 restaurants. Our search yielded the following distribution: Bronx (n = 34), Manhattan (n = 55), Staten Island (n = 7), and Brooklyn (n = 44). We obtained addresses and contact information for each restaurant and subsequently called them to verify that they each sold the Caesar salad with grilled chicken. The restaurants were matched to recent United States Census data and rank ordered by median income (United States Census Bureau, 2013). We selected a sample of 70 restaurants that were proportionate to the number of food chain restaurants in each of the four NYC boroughs of interest. We then ensured an equal number of restaurants in each of the two income-level groups (n = 35 per group).

### 2.3 Data Collection Process

Between April and June of 2013, the study’s authors collected data from the 70 selected food chain restaurants in NYC. Each researcher visited between 19 and 30 restaurants (C.H.B. = 30, D.E. = 21, S.R. = 19). A coding sheet was created and pilot-tested based on visits to five food chain restaurants not included in this sample. The coding sheet provided a systematic way of collecting and organizing data at each restaurant.

### 2.4 Variables

#### 2.4.1 Price

At each restaurant, we purchased a Caesar salad with grilled chicken and verified the pre-tax price via sales receipt. It should be noted that the Caesar salad was identified as the healthiest meal item on the current food chain menu because it is lower in calories, saturated fat, trans fats, sodium, and sugar in comparison to other menu items. In 20% of the restaurants (n = 14), we purchased a comparably priced, but less nutritious item (a popular item best described as two stacked cheeseburgers with extra condiments) to document potential variation in pricing across stores and between the two menu items. This comparison product was chosen because it is higher in calories, saturated fat, trans fats, sodium, and sugar than the Caesar salad with grilled chicken. Further, the comparison product is one of the most appealing items on this restaurant’s menu, with over 550 million sold in the U.S. alone each year (CBS, 2009).

#### 2.4.2 Promotions

Data on the number and type of promotions on the windows of each restaurant (facing outside) were collected. Specifically, each researcher noted the details of every promotion (food and/or beverage product advertised and price).

#### 2.4.3 Nutrition Information

The presence and location of nutrition information beyond calorie counts in every restaurant was recorded on the coding sheet as well.

### 2.5 Human Subjects Approval

This study was deemed exempt by the Institutional Review Boards at William Paterson University, Lehman College at The City University of New York, and Teachers College, Columbia University.

## 3. Results

Due to the extent of data collected and to assist with ease of variable organization, all data were cleaned, warehoused, and subsequently analyzed in SPSS (version 20.0). The results are presented in detail below.

### 3.1 Price: Meal Item Cost Comparison

The mean cost of the Caesar salad with grilled chicken was found to be similar across the two income-level groups: $5.37 [SD = $0.23] in the low-income neighborhoods and $5.30 [SD = $0.26] in the high-income neighborhoods. An independent sample t-test verified that this price difference was not statistically significant (t = 1.172, p = .245). The price difference between the Caesar salad with grilled chicken and the aforementioned stacked cheeseburger comparison product was similarly computed and we verified that the price differences at the restaurants were consistent across the entire sample and also across income levels (t = .435, p = .671).

The results of a one-sample t-test confirmed that, across the entire sample, the cost of the Caesar salad with grilled chicken was statistically significantly higher than any of the meal items specifically available on the reduced cost menu (t = 146.9, p < .001). Specifically, the mean cost differential between the Caesar salad and items on the reduced cost menu was found to be $4.33 (95% CI: $4.27, $4.39). Further, we verified that the nutrition content of the salad is also of higher quality than items on the reduced cost menu (see Table 1). There were three lunch/dinner meal items on the reduced cost menu at the time of the study: the 1) a cheeseburger with grilled onions, 2) a double cheeseburger with extra condiments, and 3) a breaded chicken sandwich. The nutrition quality of the Caesar salad with grilled chicken was compared to these three items. The sodium intake for the three items on the reduced-cost menu ranged from 660 - 850 mg per item in comparison to the 580 mg of sodium found in the Caesar salad. Similarly, the mean saturated fat content (5.7 grams) was found to be higher for the items on the reduced-cost menu than the Caesar salad (3.0 grams). In addition, the Caesar salad with grilled chicken was also lower in calories and sugar in comparison to its reduced-cost menu meal counterparts. Lastly, the Caesar salad comprises fewer processed ingredients than the meal items on the reduced cost menu. It should be noted that this component of the study focused on items that comprise a full meal. Side dishes and beverages were therefore excluded when comparing prices and nutrition quality. Further, since the healthiest menu item identified (Caesar salad with grilled chicken) is only offered during lunch and dinner, the comparisons also exclude breakfast items.

**Table 1:** Nutrition information and pricing: reduced cost menu meal items versus Caesar salad with grilled chicken

Description of Meal Item	Calories	Saturated Fat (g)	Trans Fat (g)	Sodium (mg)	Sugar (g)	Price
Cheeseburger with Grilled Onions	310	6	.5	660	7	$1.00
Double Cheeseburger with Extra Condiments	390	8	1	850	7	$1.00
Breaded Chicken Sandwich	360	3	0	800	5	$1.00
Caesar Salad With Grilled Chicken (Mean price reported, N = 70)	190	3	0	580	5	$5.33 (SD=$0.24)

### 3.2 Promotions

Across the entire sample (n = 70), 227 promotions were observed in the windows of the restaurant (facing outside). The number of promotions per restaurant ranged from 0 - 9, with the majority of the restaurants (91.4%) having at least one promotion. It should also be noted that multiple products were often advertised within one promotion. Although not a statistically significant difference, the low-income restaurants had more promotions (mean = 3.71, SD = 2.34) than the high-income restaurants (mean = 2.85, SD = 2.25). The frequency of each promotion across the sample was noted and the five most frequently observed food and drink promotions and their corresponding nutrition information are presented in detail in Table 2.

**Table 2:** Nutrition information for the most frequently promoted food and drink items

Item Description	Frequency (N)	Promotion	Calories	Saturated Fat (g)	*Trans* Fat (g)	Sodium (mg)	Sugar (g)
Description of Food Items							
Egg and Sausage Sandwich on a Biscuit	28	2 for $3 (n = 22)	510	14	0	1170	2
Cheeseburger with bacon and a spicy sauce	22	2 for $4 (n = 6)	610	13	1.5	1180	10
Description of Drink Items							
Fruit smoothie with blueberry and pomegranate*	15	$2.99 for a small 12 (fl oz)	220	0	0	40	44
Fruit smoothie with strawberry and banana*	15	$2.99 for a small 12 (fl oz)	210	0	0	50	44
Fruit smoothie with mango and pineapple*	15	$2.99 for a small 12 (fl oz) smoothie	210	0	0	50	44
Frozen coffee shake with caramel	15	Frappes/Shakes/Smoothies	450	12	1	125	57
Frozen vanilla ice cream shake	15	Frappes/Shakes/Smoothies	530	10	1	160	63

* These beverages were pictured together on one promotional sign

It is estimated that daily caloric intake for men and women aged 31 - 50 years should be 2200 kcal and 1800 kcal per day, respectively (United States Department of Agriculture, 2012). Saturated fat consumption should comprise less than 7% of total daily calories and trans fat should comprise less than 1% (American Heart Association, 2013a). This translates to men consuming no more than 17 grams of saturated fat and women no more than 14 grams of saturated fat per day. The Centers for Disease Control and Prevention (2013) suggests that healthy adults limit sodium intake to 2300 grams or less per day and the American Heart Association similarly suggests limiting sugar to 45 grams a day for men and 30 grams a day for women (American Heart Association, 2013b). High levels of sugar, sodium, and fat have each shown to contribute directly to the obesity epidemic and related metabolic conditions (Bleich, Wang, Wang, & Gortmaker, 2009; Baudrand et al., 2013). We found that the food items most frequently promoted at our sample of food chain restaurants to be high in sugar, sodium, and fat. Specifically, the sodium intake for the food items most frequently promoted ranged from 1170 - 1180 mg per item, comprising a significant portion of the recommended sodium intake per day for adults. Similarly, the saturated fat content was equally high for the food items most frequently promoted, ranging from 13 - 14 grams per meal item. The most frequently observed promotion was for two egg and sausage sandwiches on a biscuit, though the nutrition information available was presented for one sandwich. Should food chain customers purchase and consume both sandwiches as advertised, they would be consuming 1020 calories, 28 grams of saturated fat and 2340 mg of sodium in one meal alone. The nutrition quality of the promoted beverages, typically considered an addition or supplement to meal items, was found to be equally concerning. Of the five drinks most frequently promoted (Table 2), 100% exceeded the recommended daily amount of sugar for women, 80.0% (n = 4) contained nearly all (97.8%) the recommended daily sugar amount for men, and 40.0% (n = 2) exceeded the daily sugar recommendation for men.

### 3.3 Availability of Nutrition Information

Caloric information, per NYC’s 2008 policy to include that information on menu boards in all chain restaurants (NYC.gov, 2008), was noted at every (100%) food chain in the study sample. Additional nutrition information beyond calorie counts was available at 82.9% (n = 58) of the sample, however, this information was primarily only available after purchasing decisions had been made. Specifically, among the locations where additional nutrition was available, the majority (79.3%, n = 46) had more comprehensive nutrition information available only on tray liners, which were provided to the consumers after purchasing their food. The remaining locations had nutrition information available in a pamphlet on the wall (often located behind the register and not easily accessible by the customer), and 17.1% (n = 12) of the sample had no nutrition information available at all. In some cases, the information available was also inconsistent with calorie information included on the promotions.

## 4. Discussion

The findings of this study offer insight into three factors (price, availability of nutrition information, and promotions) in fast food settings throughout NYC. With regard to price, we found a significantly high mean cost differential between the healthiest meal item compared to items available specifically on a reduced cost menu for one dollar (US$1.00). We also determined that the most frequently promoted food items had poor nutritional quality, not aligned with current federal nutrition dietary recommendations for healthy adults. Lastly, we demonstrated that comprehensive nutrition information, when available, is primarily only available after purchasing decisions have been made.

Our findings on promotions were similar to other studies that indicate there is typically more advertising in lower-income areas (Grier & Kumanyika, 2008; Yancey et al., 2009). The items most commonly promoted tended to be high in sodium, saturated fat, and sugar. We therefore suggest that promotions in food chain restaurants focus on healthier menu items instead of nutrient-poor and calorie-dense meals and beverages. We also recommend that the promotion of healthier food items should include comprehensive nutrition information to better inform consumers prior to making purchasing decisions.

The potential impact of the calorie posting legislation is great, particularly since large numbers of individuals are currently reached by this effort (Stran, Turner, & Knol, 2013). However, given the mixed impact of these policies on improving consumer food choices, we hypothesize that comprehensive nutrition information, beyond just calories, is needed. Comprehensive nutrition information beyond calorie counts was difficult to locate in all the food chain restaurants visited. Information was typically available on the back of tray liners for those who ate in the restaurant and was given to the customer only after the food was ordered. Providing customers with more visible and readily accessible comprehensive nutrition information may positively impact point-of-purchase decisions.

While it is noteworthy that this particular food chain is making healthier menu items available, further exploration into the attainability of these items due to cost warrants further investigation, particularly since studies have suggested that customers, especially those in low-income neighborhoods, tend to purchase and consume foods that are priced more affordably (Gordon-Larsen, Guilkey, & Popkin, 2011). The significant cost differential between the healthier food items on the food chain menu and those specifically available on the reduced cost menu is concerning. Given that low-income neighborhoods in NYC are disproportionately affected by the obesity epidemic (Kim, Berger, & Matte, 2007; New York City Department of Health and Mental Hygiene, 2013), the need to make healthier food items more affordable at fast food restaurants is critical. In light of these findings, we suggest that fast food companies enhance their efforts to promote better nutritional choices among their customer base by exploring the price reduction of more healthful items on the menu. Indeed, studies of adolescent youth have shown that pricing impacts both body mass index (BMI) (Powell & Bao, 2009; Powell, 2009) as well as the likelihood that fruits and vegetables will be consumed (Powell, Auld, Chaloupka, O’Malley, & Johnston, 2007; Beydoun, Powell, & Wang, 2008).

On an international level, the global fast food restaurant industry is growing rapidly (QSR, 2012). Such chain stores can be found in several countries around the world and are part of a shift in eating patterns noted by public health professionals (Hollands, Campbell, Gilliland, & Sarma, 2013; Meetoo, McGovern, & Safadi 2007) Although the menu offerings in these chain restaurants can vary slightly given cultural context, the nutritional value of fast food is often compromised by the processed nature of these foods, their lack of important nutrients, and the ways in which they are prepared. We suggest that studies such as ours be replicated internationally in order to gain a greater understanding of this food environment with its growing global reach.

## 5. Conclusion

This descriptive study is limited in that the data were collected at one time point and therefore only provides us with insights during that specific time frame. In addition, we looked at only one fast food chain; there are many others in the NYC area that could have other marketing, pricing, and nutrition information practices. The implications of this work are nonetheless significant, particularly given high rates of obesity in NYC and in low-income neighborhoods. Efforts to address the obesogenic nature of these fast food environments in NYC and elsewhere may contribute to lowering rates of obesity and chronic disease in NYC and other communities. Further, given that children and adolescents who regularly frequent fast food restaurants may be at increased risk of becoming obese (Bowman, Gortmaker, Ebbeling, Pereira, & Ludwig, 2004), the need to promote healthier food choices and increase the accessibility of nutritious foods is of utmost importance.
